# Exploring womens’ experiences and decision making about physical activity during pregnancy and following birth: a qualitative study

**DOI:** 10.1186/s12884-019-2707-7

**Published:** 2020-04-16

**Authors:** Amy Findley, Debbie M. Smith, Kathryn Hesketh, Chris Keyworth

**Affiliations:** 1 Manchester Centre for Health Psychology, Division of Psychology and Mental Health, School of Health Sciences, Faculty of Biology, Medicine and Health, Manchester Academic Health Science Centre, The University of Manchester, Coupland 1 Building, Room G.3, Oxford Road, Manchester, M13 9PL UK; 2 School of Social and Health Sciences, Leeds Trinity University, Leeds, England; 3 MRC Epidemiology Unit and Centre for Diet and Activity Research, University of Cambridge, Cambridge, UK; 4 UCL Great Ormond Street Institute of Child Health, London, UK

**Keywords:** Physical activity, Pregnancy, Decision making, Postpartum, Qualitative, Thematic analysis

## Abstract

**Background:**

Physical activity (PA) tends to decline during pregnancy, and remains low in the early postpartum period, despite the known physical and psychological benefits. This study aimed to explore: (1) women’s experiences of PA during pregnancy and following birth; and (2) decision-making processes related to PA during this time.

**Methods:**

Semi-structured telephone interviews were conducted with 16 women who were either pregnant or had recently given birth. Interviews were transcribed verbatim and analysed using thematic analysis.

**Results:**

There were two over-arching themes: (1) ownership of body, which consisted of the sub-themes: others try to take ownership, important to maintain fitness into pregnancy and motherhood, expectations of PA, and pressure to conform; and (2) unknown territory, which consisted of the sub-themes: engaging in PA with caution, and unclear advice. Decision-making about PA during pregnancy was influenced by: pressure from others who felt responsible to protect the woman from coming to harm; pressure from social media to lose weight postpartum; participant’s beliefs about the benefits of maintaining fitness and participants expectations of how active they thought they would be able to be during pregnancy. Participants felt that pregnancy was an ‘unknown territory’ in terms of the unfamiliar feelings in their body and the inability to continually monitor their baby for reassurance of baby’s health. Advice received from midwives was often lacking, or not tailored to the individual. Advice from friends and family was often regarded as incorrect, but still caused doubt and fear of PA during pregnancy.

**Conclusions:**

These findings contribute to the understanding of women’s experiences of PA during pregnancy and post-partum, and their decision-making processes about PA during pregnancy. Developing accurate and tailored advice as part of midwifery care, that considers the physical and psychological aspects of engaging in PA during pregnancy, will help to ensure that women are supported to make informed decisions about their PA behaviour.

## Background

The Department of Health recommends that pregnant women engage in 150 min of moderate intensity physical activity (PA) per week [1]. Regular PA during pregnancy is beneficial to physical and psychological health, improving cardiovascular and muscular health and reducing the risk of insomnia, anxiety and depression [2]. Regular PA during pregnancy has also been linked with shorter length of labour and reduced risk of complications during delivery [2]. The Royal College of Obstetricians and Gynaecologists (RCOG) recommend that PA during pregnancy is safe, although women should not engage in any new activity; they should avoid activities in environments with hot temperatures or high altitudes; and activities that involve risk of falling or being hit in the abdomen [2].

Despite recognised public health guidelines encouraging PA during pregnancy, PA levels often decline during pregnancy, particularly during the second and third trimesters, and remain low until at least three months after birth [3], or completely cease [4]. A possible reason for this decline is that women are more likely to change the type of PA they engage in (i.e. switching to lower intensity activities which may not provide as many health benefits [3]). Women may also cease their PA as a result of their lived experiences of being pregnant and/or their perceptions of possible risk to the fetus [4].

The United Kingdom’s (UK) Chief Medical Officers recommend that research should explore the beliefs about PA amongst pregnant women, including the barriers and enablers to PA [5]. Understanding women’s experiences of PA during pregnancy and postpartum is important to help encourage PA. This is especially true given that pregnancy is often regarded as a “teachable moment”: women may be more open to adopting health behaviours as a result of pregnancy [6]. It is also a time where pregnant women are in regular contact with health providers who are in a position to support health behaviour change with their patients [6, 7]. Indeed, for many women, pregnancy may be the only time that they engage with health providers.

It is therefore important to understand the specific influences on women’s decision-making processes regarding PA during pregnancy. A recent systematic literature review examining PA during pregnancy [8] highlighted pregnancy-specific barriers including physical limitations, the presence of health conditions, tiredness, pain, a lack of motivation to engage in PA, lack of self-confidence and a lack of time to engage in PA. In addition to these barriers, the review found that women reported a lack of knowledge regarding the type of PA they could safely engage in during pregnancy. These studies support the understanding of the barriers involved in engaging in PA during pregnancy, but importantly, they highlight a lack of clear, well-defined healthcare professional guidance. There is a lack of qualitative research investigating the decision-making processes involved in engaging in PA during pregnancy.

Understanding where women get advice about PA during pregnancy and what advice they act upon is necessary for improving the healthcare support provided. A recent study in the United States of America (US) found that women most commonly reported receiving information about PA during pregnancy from books, the internet, and healthcare professionals and were most likely to follow advice received from a doctor, nurse or dietician [9]. Another recent study found that midwives in the UK perceive being subjected to increasing demands and expectations from pregnant women to advise on PA recommendations during pregnancy [10]. It is crucial that healthcare professionals provide the right guidance and support to women during pregnancy to ensure they can be confident that they are engaging in safe levels of PA. The National Institute for Health and Care Excellence (NICE) advise that healthcare professionals should discuss PA during pregnancy at the earliest opportunity to address: (a) any concerns that women may have; and (b) to advise them of the benefits of PA for themselves and their baby [11]. However, whilst a recent survey of midwives in the UK showed a large number of midwives reported that they were confident in answering questions regarding knowledge of the NICE guidelines for PA during pregnancy, a much smaller proportion of respondents correctly identified the PA guidelines [12].

To address the limitations of the current evidence base, this study used qualitative methods to explore and develop an understanding of: (1) women’s experiences of PA during pregnancy and following birth; and (2) decision-making processes in relation to engaging in PA during this time.

## Methods

### Design and setting

Semi-structured interviews were conducted to explore women’s views and experiences relating to PA during pregnancy and postpartum. Interviews were conducted by telephone, with nulliparous pregnant and postpartum women from the north of England. Ethical approval was gained from The University of Manchester research ethics committee (Ref: 2017–0945-1936). Written informed consent was obtained before the interview took place, and consent was confirmed verbally at the beginning of each interview.

### Sample characteristics

Women were eligible to take part if they were aged 18–40 years old, lived in England and spoke fluent English. Participants were either currently pregnant for the first time or had given birth to their first child less than three months before the recruitment date, as this is when PA has been shown to decline [3]. Participants were of a healthy weight (self-reported pre-pregnancy body mass index of 18.5 to 25 [13]). We focused on women of healthy body mass index, as this group are often overlooked in research studies, which tend to focus on PA levels in pregnant women who are overweight or obese. Participants were ineligible if they had a medical issue that prevented them from being physically active, or if they had been previously pregnant (including previous miscarriage or stillbirth), as these women’s experiences and knowledge has been shown to influence how they understand and engage in PA [14].

### Procedure

A purposive sampling method was used for recruitment, which was carried out through online advertisements, social media groups and email distribution lists at a large North-West university. Participants were recruited until no new ideas were evident in the interviews (*n* = 16), determined by consensus amongst the research team. Written and verbal consent was obtained from each participant prior to their interview. Telephone interviews were suitable in this population as they were less burdensome for participants who may have found it hard to find the time to attend a face-to-face interview whilst pregnant, or as a new mother [15]. An iterative topic guide was developed based on previous literature and expertise within the research team (Table 1). A group consensus was achieved on the topics included within the interview topic guide and the guide was piloted on a new mother.

**Table 1:** Interview guide questions

Topic	Questions and prompts
About you	
	Q. Could you tell me about your current pregnancy status and tell me a bit about how your pregnancy has been so far?
	Prompt: What stage are you at in your pregnancy/ post pregnancy?
	Q. How would you describe your PA levels?
	Prompt: What did you expect your PA levels to be like once you got pregnant?
	Prompt: Did your PA change between trimesters?
Views on PA during pregnancy	
	Q. What are your thoughts on PA during pregnancy?
	Prompt: Do you think it is safe/ risky?
Costs and benefits of PA	
	Q. What are your views of the costs and benefits of doing PA whilst pregnant?
	Prompt: Do you feel the costs outweigh the benefits/ benefits outweigh the costs?
Sources of information	
	Q. Could you tell me about where you have got most of your information from concerning PA whilst pregnant?
	Prompt: What sort of advice was this?
	Prompt: How did it make you feel about PA during pregnancy?

All interviews were conducted by the first author (AF), who received prior training in conducting qualitative interviews. Interviews were recorded using a password-protected voice recorder and were transcribed verbatim, using a pseudonym to identify each participant. Participants received a high street shopping gift voucher for taking part.

### Analysis

An inductive and deductive approach was taken when analysing the interviews. The themes were data driven, however, as the interview guide was based on previous literature, relevant prompts were used to guide the interview [16]. A realist approach to analysis was taken to explore the women’s views and experiences of PA. This meant accepting the data at face value and assuming that the participant’s reports were a true reflection of their experiences [16]. The research team adopted this approach by interpreting quotes literally and not questioning whether participants’ statements were a misinterpretation of the true event. Themes were identified at the semantic level, analysing the explicit meanings of the data [16]. The six stages of thematic analysis, described by Braun and Clarke [16], were used to analyse the data (see Table 2 for a description of the methodology). NVivo version 11.1.1 was used to analyse the interview data and extract relevant quotes. The consolidated criteria for reporting qualitative research (COREQ) checklist [17] was followed.

**Table 2:** The six phases of thematic analysis as described by Braun and Clarke [6]

Phase	Description
1. Familiarisation with the data	Familiarisation was done independently by AF whilst conducting the interviews and transcribing the audio recordings. AF wrote interview summaries after each interview and kept a diary to note any thoughts and reflections down during the interview stage of key topics that had been emerging. All authors familiarized themselves with the transcripts by reading the transcripts.
2. Generating initial codes	A coding process was implemented whereby data that was relevant to the research question was highlighted and assigned a code. The codes were then applied to further pieces of data that corresponded to the same concept. This was conducted by all authors on two transcripts selected at random by AF. Once the authors had individually derived a code lists for the first two transcripts, code lists were compared between authors and any discrepancies between codes were resolved by consensus. Phase 2 was then completed by AF, DS and KH on the remaining transcripts. The coding was done in a systematic and iterative way across the data whereby any new codes were consistently coded across the rest of the data when that concept occurred.
3. Searching for themes	AF, DS and KH individually reviewed and grouped codes according to similarity of topic and started to form potential themes. The authors individually developed theme names that captured all codes included within the theme. AF, DS and KH cross checked each other’s themes to ensure full agreement of the research team.
4. Reviewing themes	AF, DS and KH checked the themes in relation to the coded data extracts and assessed in terms of how well they represented the entire data set. Each author mapped their themes onto each other’s to ensure consistency and agreement across themes. AF, DS and KH discussed any discrepancies of themes to come to a consensus on the main themes to represent the data.
5. Defining and naming themes	The names and definitions of each theme were refined and a short description of each theme was developed by authors AF, DS and KH. Each author re-assessed the names of themes to ensure theme names captured succinctly the concepts relating to the theme. Specific contributions of the data were pinned down to clarify the story of the analysis.
6. Producing the report	A full report of the analysis was written by AF with the use of the most appropriate extracts and relating back to the original research question. Guidance was received from CK, DS and KH.

## Results

**Fig. 1:**
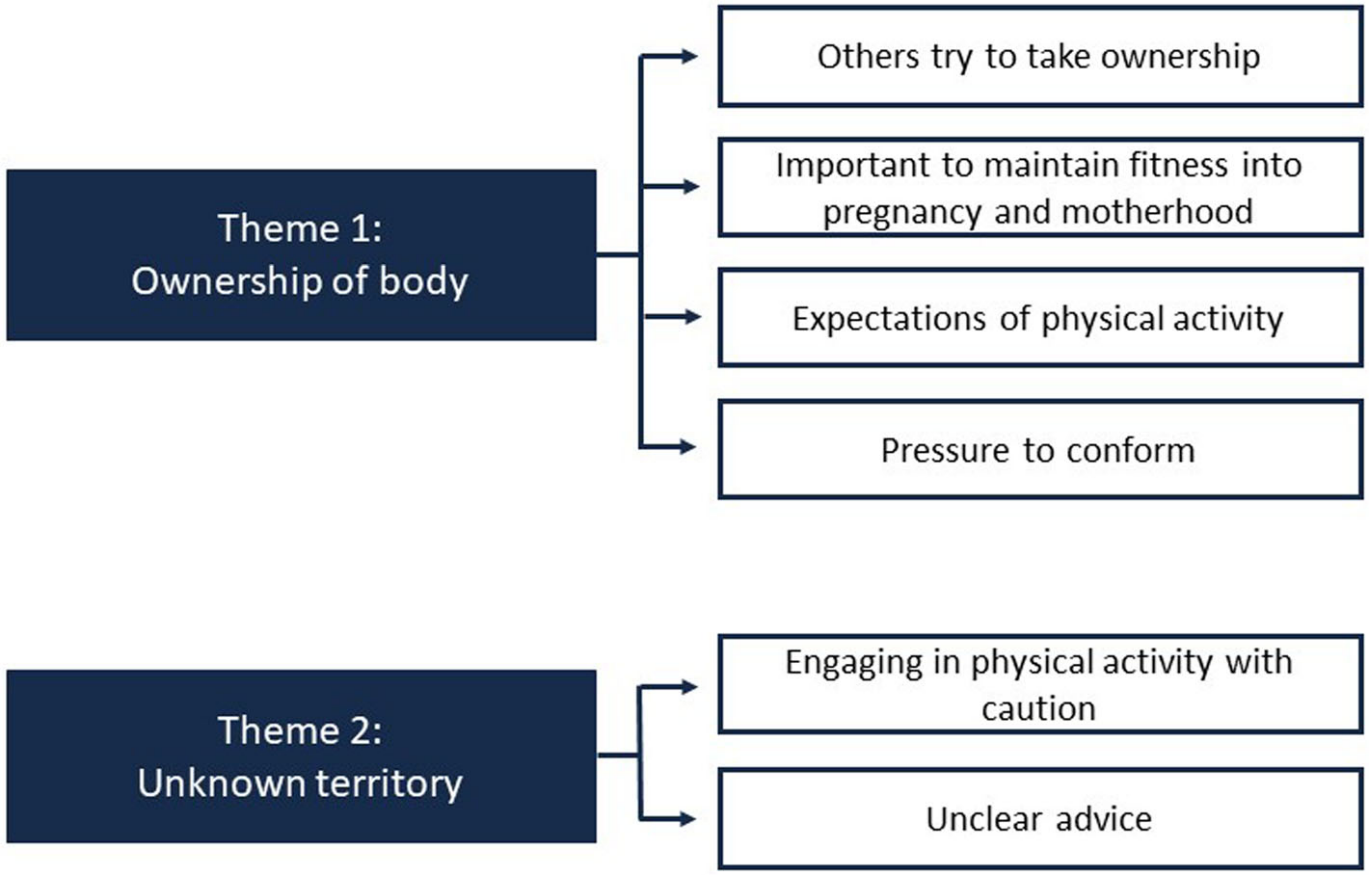
Thematic map of key themes and sub-themes

Sixteen participants took part in the study; 11 were currently pregnant and five had given birth within the past three months. Pregnancies ranged from 14 to 38 weeks (*M* = 25.7 weeks, *SD* = 8.7). No participants were in their first trimester. Out of the five participants who had already given birth, the women ranged from three to 13 weeks post-partum (*M* = 7 weeks, *SD* = 3.7). The interviews lasted between 32 and 80 min (*M* = 54.1 min, *SD* = 13.0). Results are presented according to the two over-arching themes (see Fig. 1): *ownership of body* and *unknown territory*. Verbatim quotes from the interviews are used to support these themes.

### Theme 1: ownership of body

Participants perceived PA as a personal experience, and all were aware of the perceived benefits of PA for themselves and their baby. Most participants reported that whilst other people had tried to offer advice, they found it important to listen to their own body. Pregnancy-specific physical symptoms that were not usual for the women made it hard for some participants to feel that they knew and thus owned their body. Familiarity of new physical and psychological feelings increased with gestation which allowed women to feel like they knew their bodies again The ownership of their body and the personal choice of PA was illustrated with four sub-themes: others try to take ownership; important to maintain fitness into pregnancy and motherhood; expectations of PA; and pressure to conform.

#### Others try to take ownership

Most participants reported that others offered unsolicited advice, leading them to feel a lack of ownership over their own bodies.

*“Most people say you should be resting you shouldn’t be doing much but I stayed quite active because I felt fine, I don’t want to be resting all the time I want to carry on with my day to day life I don’t really feel much different other than having to be more, I do still feel good in myself like I’ve got plenty of energy etcetera*” (Molly).

There was also a lack of trusted information available, with many people (including family and partners) advising women that PA should be stopped during pregnancy, resulting in most participants finding it difficult to know which advice to follow. Changes to work-based PA, such as lifting and moving heavy objects, were reported by around a quarter of the participants. This was often influenced by others at work, despite the participants feeling confident that they would be fine to do the activity themselves.

*“Yeah so, I don’t know, the stigma is terrible, a lot of the time people just don’t want you to do anything. I think it’s really old fashioned isn’t it. Like in the olden days pregnancy you sat with your feet up and relaxed for 9 months”* (Sally).

It was evident in around half of the participants’ accounts that changes in PA levels during pregnancy reflected pre-pregnancy PA levels as participants who discussed being more active pre-pregnancy were more likely to be active during pregnancy. This demonstrates that in the case of suggested PA levels, there is ‘no one size that fits all’ and personal ownership becomes more important. Most participants believed they understood the feeling of their body pre-pregnancy, but their changing pregnancy body often felt unfamiliar, leaving around half of the participants feeling like they no longer had control over it.

*“I think it means that I don’t necessarily know my body and my health in relation to pregnancy as I did pre-pregnancy so, it’s something that I’ve never experienced before so I don’t know, I don’t know how it’s going to affect my ability to do things”* (Jane).

#### Important to maintain fitness into pregnancy and motherhood

Most participants reported that being healthy was important during pregnancy, and as such were determined to continue with their healthy behaviours (including a healthy diet and engaging in physical activity). Physically, some participants felt muscle strength and breathing techniques had improved; a few reported that they were in control of their weight gain; others reported that their aches and pains were reduced; and a couple of participants felt more energised. Psychologically, some participants described how PA had helped lift their mood and made them feel relaxed. Being physically active made a few participants feel mentally and physically prepared for labour, as well as around a quarter of the participants acknowledging the impact on their baby’s physical health.

*“I felt mentally very prepared because of the exercise I did [ …*] *it [labour] was quite overwhelming but I felt confident in my strength so even if I felt like I couldn’t do it I was prepared as I could be to do it so I try to just think of that and that seemed to work”* (Susan).

All participants reported a decrease in PA during or before the final stages of pregnancy. Certain activities that were regarded as too intense by some participants, such as cycling or running, were often swapped for activities perceived to be less intense and assumed to be safer, such as yoga or swimming. A few participants reported that the tiredness of their body meant that they needed to reduce their PA levels.

*“No I mean I feel a bit more tired than before that’s the only thing like before I could do 3 hours and be fine, now if I do the 3 hours I feel I need to sit down at some point and also because now the baby is kicking more and moving more sometimes she puts herself into positions where it feels less comfortable so you have to sit down or stretch or something, I feel that why now I’m doing yoga for pregnancy now instead of my cycling or whatever*” (Jennifer).

Side effects of pregnancy, such as morning sickness, fatigue, breathlessness, and aches and pains, created physical limitations that restricted some participants activity levels, and were sometimes perceived as signs that the women were pushing themselves too hard, potentially causing harm to themselves or their baby.

*“So I think it (feeling queasy and out of breath) makes you more aware that you can’t push yourself as you would have done but I think that’s a good thing because your body’s saying, OK don’t push yourself as much”* (Jane).

#### Expectations of physical activity

The participants’ pre-existing expectations of the level of PA they would be able to achieve during pregnancy were discussed as being influenced by several factors. These included; knowledge of how active other pregnant women had been during pregnancy, and advice provided by healthcare professionals. Around a quarter of participants reported that they were more active during their pregnancy than they had expected to be. Two participants reported that they were less active during their pregnancy than they had expected to be.

*“The running community people were very supportive and actually quite confident I could do that, but other people weren’t so keen, like other non-active people you know they would think oh is that safe and so on, yeah so if your not into, I think active people would say yes I think your doing OK keep doing it and non-active people would say oh are you sure you should be doing it?”* (Charlotte).

Expectations that other people had of how active the women should be during pregnancy also varied depending on that person’s own experiences. Two participants believed that compared to inactive people, those who were active had a different expectation of PA during pregnancy. Around a third of participants reported that active people were more likely to be supportive of PA during pregnancy, and inactive people to be less supportive of PA during pregnancy.

#### Pressure to conform

Most participants reported feeling a strong sense of social pressure to conform to other people’s views of PA during pregnancy. Participants often compared themselves to others and recalled listening to stories from others who had, or had not, continued PA during pregnancy, which influenced their perceptions. Likewise, social media created pressure for a few participants to regain levels of PA following pregnancy and to enable their bodies to return to similar levels of their pre-pregnancy fitness.

*“I think the pressure of it makes you feel guilty really because I thought oh maybe you see all these women you know who just bounce straight back and like there’s a lot of people on Instagram and stuff like that”* (Sally).

A few participants reported that they had felt they have had a ‘lucky’ pregnancy, feeling relatively similar in their body compared to during pre-pregnancy. The view of other people, that pregnant women should not engage in PA had a strong impact on some participants, with some participants reporting that it is easier to comply with this view than feel bad about being active.

*“I felt more justified that I should be taking a rest. So I think probably I definitely, probably it made me do less but also the fact that I was already doing a bit less made me not feel as bad about it so you know kind of like made me feel like it really is the right thing to do because I need to look after myself*” (Jane).

### Theme 2: unknown territory

Most participants felt that they were entering unknown territory, as they were experiencing a conflict between wanting to be physically active, but also wanting reassurance that the baby was healthy. The lack of being able to monitor baby’s health created worry for most participants as this made PA feel like a risky behaviour. This theme has two sub-themes: engaging in PA with caution; and unclear advice.

#### Engaging in PA with caution

Most participants felt some level of uncertainty because they had no way of knowing if or how their baby would be affected by PA. Scans or movement from the baby provided some reassurance for most women that their baby was healthy. However, as these scans were sparse, over half of participants reported feeling anxious between scans generally, and reported that this meant that they were not able to confirm whether PA was causing harm to their baby, which increased their sense of risk of PA.

“*That scan its only so many weeks apart, you don’t know the immediate impact of activity on the baby, you know for instance how you know if you go for a run does it impact the baby’s heart rate, well there’s no way to know because nobody monitors it”* (Charlotte).

Physical triggers, such as a change in their body shape, made it more salient to around a quarter of participants that they were carrying a baby. This led to a few participants re-considering their PA levels, as they did not want to ‘bump’ their baby while it developed.

*“So I am still doing as much as I was before but just not doing as high intensity, maybe like brisk walking and swimming rather than the gym and bike riding like I did before [ …*] *I was getting back ache and stuff so I didn’t think it was a good sign, I didn’t know how it could link to the baby specifically but I just sort of avoided it [bike riding] afterwards”* (Molly).

Most participants reported that they would not be able to forgive themselves if something happened to their baby, which lead to a fear of PA in a few participants. Around a quarter of participants reported that reducing or stopping PA allowed them to be more cautious, which made them feel more comfortable, given the lack of reassurance they received about their baby’s health. A couple of participants stopped PA altogether as soon as they found out they were pregnant, even if they and their baby were in good health.

*“more because even though I know that exercise is great and its fine while you’re pregnant it was just always in the back of my mind well what if I hurt the baby I wouldn’t be able to forgive myself I’d rather miss out on a couple of months exercise to make sure that my baby’s fine”* (Amy).

#### Unclear advice

Over half of participants gained variable information from healthcare professionals during pregnancy and following birth. Most participants expressed that they wanted advice that was either evidence-based or was from professionals who were trained to know what constitutes safe activity while pregnant. Around half of participants reported that the advice they received was unclear and/or conflicting in nature. The conflicting information left some participants feeling alone in knowing what to do, which ultimately left them to rely on their ‘common sense’. For example, one participant was told by her General Practitioner (GP) that she should not run again until after 12 weeks post-partum, whereas her friend who was also pregnant was told by a different GP that she would be OK to start jogging again at six weeks post-partum. Knowledge of PA guidelines during pregnancy was also variable among the participants. Generally, the few participants who had good knowledge of the PA guidelines described themselves as being more physically active than those who did not (although physical activity levels were not assessed). Most participants often sought information for themselves by searching the internet, which again often lead to varying/contradicting information which was liable to be misunderstood.

*“I had very different advice from my GP to a friend who had a similar delivery in terms of when you can start exercising again so I think generic advice was helpful but I think it can be quite variable in terms of the individual that you actually speak to”* (Michelle).

A couple of participants perceived the advice to be overly cautious, as a result of their healthcare professional or sports trainer feeling personally liable if anything harmful happened to the woman or her baby while she was in their care. This meant that participants felt like the advice they received was not tailored to their personal circumstances and was more risk averse than necessary. As a result, the advice was consequently ignored by a few participants.

*“Your like GP or midwife are going to be cautious, they’re going to be on the safe side and they’re going to tell you well just don’t do too much physically”* (Charlotte).

## Discussion

In examining women’s experiences of PA during pregnancy and following birth, and their decision-making processes in relation to engaging in PA during this time, two themes of ‘ownership of body’ and ‘unknown territory’ emerged. Theme 1, ‘ownership of body’, can be understood in relation to: others trying to take ownership, importance of maintaining fitness into pregnancy and motherhood, expectations of physical activity and pressure to conform. Theme 2, ‘unknown territory’ pertains to women engaging in physical activity with caution, and receiving unclear advice from external sources.

Firstly, the theme of ownership of body was discussed by most participants in relation to other people trying to take ownership over their PA behaviour. This resonates with research on benevolent sexism where people feel obliged to ‘protect’ pregnant women from coming to harm, and as such act in a way to restrict pregnant women’s health behaviours, albeit with good intention [18, 19]. This unsolicited advice appeared to have impacted women’s decision-making processes, as women felt that they were not ‘allowed’ to do certain activities while being watched by others who disapproved.

Secondly, some participants described a pressure to get back into shape quickly post-partum, often referencing specific influential people on social media. The negative effects of media on women’s body image while pregnant has been explored by a recent qualitative study, finding that nearly half of participants expressed feeling negatively about their body due to pregnancy and/or postpartum media images [18]. Most of the women in the media study felt pressure to lose weight quickly after giving birth and guilt if they did not; similar perceptions were held by some of the participants in this study.

Participants were unable to continually monitor the health of their baby between routine scans which left them to rely on their symptoms for indications of the baby’s health. Previous research has found that pregnant women find reassurance from antenatal scans that their baby is healthy [20]. Consistent with previous studies [14, 21–23], participants perceived symptoms such as fatigue, nausea, and pain, to be linked with increased risk to their baby, and therefore altered their PA behaviour to try and compensate for the perceived threat. Our findings suggest that participants felt responsible for the health of their baby [14, 24], and consequently modified their activity levels by reducing the amount or intensity of PA to protect their baby from any possible risk [14, 21, 22].

A decline in PA during pregnancy has been frequently reported in the literature [3, 25, 26], and it was a key aspect of this study to understand how women’s perceptions of PA during pregnancy could contribute towards this decline, rather than encourage maintenance of PA levels. Although some participants saw reducing their PA as a potential way to limit the risk of harm to their baby, most participants did not completely abstain from PA. They acknowledged feeling a sense of physical and psychological benefit when active, which overpowered feelings of anxiety and consequently reduced their perceptions of risk associated with PA. Similar feelings of benefitting from PA have also been reported in other studies [22, 27] and have been identified as motivating factors for participants to stay active throughout pregnancy.

Our study showed that participants felt that PA helped them mentally and physically prepare for labour [28], reducing their anxiety and being beneficial for the health of themselves and their baby, rather than a threat. Consequently, it is important that women are aware of the potential benefits of being active during pregnancy so that they can make informed decisions about their behaviour. These findings are consistent with traditional theories of risk perception [29], and how perceptions of risk are formed as a result of: [1] how a person appraises the severity of a threat, and [2] their vulnerability to a negative outcome.

Our study found that risk perceptions were formed as a result of not being able to continually check on their baby’s health between routine scans, and how vulnerable to harm they felt their baby was from their PA behaviour. These risks meant that the majority of participants engaged in PA with caution by changing the type of activity that they took part in or reducing (and sometimes ceasing) their PA levels. This has implications for intervention development to support women to be physically active during pregnancy. Future research should aim to understand the relationship between these factors and their subsequent impact on perceptions of risk, as well as actual PA levels.

How informed participants felt about engaging in PA during pregnancy influenced their decision-making processes. Understanding where women receive support and advice from regarding PA during pregnancy is vital to knowing how to develop targeted interventions. NICE [11] advise that healthcare professionals should discuss how physically active a pregnant woman is at the earliest opportunity to address any concerns women may have, and to advise them on the benefits to both herself and the baby. Likewise, the UK Chief Medical Officers produced guidelines for PA in pregnancy [30]. Despite this, most participants perceived a lack of guidance from healthcare professionals, which is consistent with findings from other studies [21, 22, 27, 31–34]. In addition, when participants did receive advice from healthcare professionals, or sports trainers, they often felt like this advice was contradictory and overly cautious. This is consistent with findings from other studies [22, 33, 35], in which participants felt the advice was not tailored to their situation.

Our study suggested participants showed a tendency to use their own judgement to decide whether an activity was “risky”, however, sometimes participants reported being too uninformed to make this decision, and consequently, their risk perceptions were heightened. Although participants often felt let down by the guidance they received from health professionals and sports trainers, they still sought advice from these people. Health professionals are both an expected and trusted source of information regarding health behaviour [36]: they therefore need to recognise their important role in providing accurate information and are able support women in regards to their PA behaviour.

### Implications for practice

These findings are important to inform maternity practice. Health, sport and exercise professionals need to recognise their influence over decision-making processes regarding PA behaviour during pregnancy and post-partum, and have a duty to provide accurate and supportive advice. This advice should be tailored to take into account women’s previous PA habits and their pregnancy experience, and should therefore ask women about their circumstances during antenatal appointments. More support could prepare women for the physical changes they will experience during pregnancy and to help them feel comfortable with their changing body, rather than perceiving the symptoms as a threat, and thus reducing their PA behaviour as a result. Women who are particularly at risk of perceiving their baby to be especially vulnerable could be identified and provided with appropriate support to overcome their worries around PA. This could be done through midwives initiating conversations about the symptoms of pregnancy and discussing what is normal and safe to feel in their body while engaging in PA.

Midwives could also provide more advice on how to self-monitor the baby’s health, such as taking note of the baby’s movements and knowing at what point they need to make contact with their midwife to raise concerns over the baby’s health. This would enable women to feel more confident in knowing how much activity is safe to do without causing harm to the baby. As recommended by NICE [11], women should be advised on the potential benefits of PA to themselves and their baby during pregnancy; however, it is apparent from this study that not all participants felt they received this.

This study has some limitations. Firstly, the sample size was small and it is possible that with a larger sample size a wider range of perceptions and experiences could have been reported. However, it was determined by the researchers that in the later interviews, no new data outside of the developed themes was being collected, and that saturation of experiences had be reached, thus suggesting that the sample size was adequate to answer the research question. Secondly, a large proportion of the participants were recruited via an email sent out through the university. Although demographic characteristics and pre-pregnancy PA levels were not recorded to maintain confidentiality, it is possible that participants were more highly educated and active than the general population, preventing wider generalisability of these findings. Previous research has found racial and ethnic differences in the amount of PA women take part in during pregnancy [37], therefore, it is possible that this sample was not a diverse sample and may not reflect a range of experiences. Similarly, while we explored PA levels during the interview, participants’ actual activity levels were not measured during pregnancy or pre-pregnancy and so it is unclear how active the sample actually was. In addition, most of the participants recruited were active pre-pregnancy which is likely to have influenced their decision-making processes on PA. Future research would benefit from obtaining views from both a wider demographic and women with varied PA levels, e.g. women who were not currently active, or those who were active before pregnancy.

## Conclusion

This study contributes to the literature through its exploration of women’s experiences of PA in pregnancy and following birth, and how their decision-making processes about engaging in PA are formed. Developing accurate and tailored advice, as part of midwifery care for example, that considers the physical and psychological aspects of engaging in PA during pregnancy, could help to ensure that women are supported to make informed decisions about their PA behaviour.

## Data Availability

The data that support the findings of this study are available on request from the corresponding author. The data are not publicly available due to privacy or ethical restrictions.

## Abbreviations

COREQ: Consolidated criteria for reporting qualitative research
GP: General Practitioner
NICE: National Institute for Health and Care Excellence
PA: Physical activity
RCOG: Royal College of Obstetricians and Gynaecologists
UK: United Kingdom
US: United States of America

## References

[1] Department of Health. Physical activity in pregnancy infographic: guidance. London;UK: Department of Health; 2017.

[2] Royal College of Obstetricians and Gynaecologists (RCOG). Exercise in pregnancy. Statement No. 4. London; UK: Royal College of Obstetricians and Gynaecologists (RCOG); 2006.

[3] Borodulin K, Evenson KR, Herring AH (2009). Physical activity patterns during pregnancy through postpartum. BMC Womens Health.

[4] Swift JA, Langley-Evans SC, Pearce J, Jethwa PH, Taylor MA, Avery A (2017). Antenatal weight management: diet, physical activity, and gestational weight gain in early pregnancy. Midwifery.

[5] Reid H, Smith R, Calderwood C, Foster C (2017). Physical activity and pregnancy: time for guidance in the UK. Br J Sports Med.

[6] Phelan S (2010). Pregnancy: a teachable moment for weight control and obesity prevention. American J Obstetrics Gynecology.

[7] Keyworth C, Epton T, Goldthorpe J, Calam R, Armitage CJ (2019). It's difficult, I think it's complicated': health care professionals' barriers and enablers to providing opportunistic behaviour change interventions during routine medical consultations. Br J Health Psychol.

[8] Thompson EL, Vamos CA, Daley EM (2017). Physical activity during pregnancy and the role of theory in promoting positive behavior change: a systematic review. J Sport Health Sci.

[9] Mercado A, Marquez B, Abrams B, Phipps MG, Wing RR, Phelan S (2017). Where do women get advice about weight, eating, and physical activity during pregnancy?. J Women's Health.

[10] De Vivo M, Mills H. “they Turn to You First for Everything”: Insights into Midwives’ Perspectives of Providing Physical Activity Advice and Guidance to Pregnant Women.”. SportRxiv May. 2019;14. 10.1186/s12884-019-2607-x PMC6891952 31795961

[11] National Institute for Health and Care Excellence (NICE). Weight management before, during and after pregnancy. Public Health Guideline. PH27. London; UK: National Institute for Health and Care Excellence (NICE); 2010.

[12] Hopkinson Y, Hill DM, Fellows L, Fryer S (2018). Midwives understanding of physical activity guidelines during pregnancy. Midwifery.

[13] Eveleth PB (1996). Physical status: the use and interpretation of anthropometry. Report of a WHO expert committee. American J Human Biology: Official J Human Biology Association.

[14] Hanghoj S (2013). When it hurts I think: now the baby dies. Risk perceptions of physical activity during pregnancy. Women and Birth.

[15] Lupton D (2000). 'A love/hate relationship': the ideals and experiences of first-time mothers. J Sociol.

[16] Braun V, Clarke V (2006). Using thematic analysis in psychology. Qual Res Psychol.

[17] Tong A, Sainsbury P, Craig J (2007). Consolidated criteria for reporting qualitative research (COREQ): a 32-item checklist for interviews and focus groups. Int J Qual Health Care.

[18] Liechty T, Coyne SM, Collier KM, Sharp AD (2018). “It’s just not very realistic”: perceptions of media among pregnant and postpartum women. Health Commun.

[19] Sutton RM, Douglas KM, McClellan LM (2010). Benevolent sexism, perceived health risks, and the inclination to restrict pregnant Women’s freedoms. Sex Roles.

[20] Roberts J, Griffiths FE, Verran A, Ayre C (2015). Why do women seek ultrasound scans from commercial providers during pregnancy?. Sociology health illness.

[21] Evenson KR, Moos MK, Carrier K, Siega-Riz AM (2009). Perceived barriers to physical activity among pregnant women. Matern Child Health J.

[22] Hegaard H (2010). K, Kjaergaard H, Damm P, P, Petersson K, dykes A-K. experiences of physical activity during pregnancy in Danish nulliparous women with a physically active life before pregnancy a qualitative study. BMC Pregnancy Childbirth.

[23] Connelly M, Brown H, van der Pligt P, Teychenne M (2015). Modifiable barriers to leisure-time physical activity during pregnancy: a qualitative study investigating first time mother's views and experiences. BMC pregnancy childbirth.

[24] Lupton D (2012). ‘Precious cargo’: foetal subjects, risk and reproductive citizenship. Crit Public Health.

[25] Cramp AG, Bray SR (2009). Pre-and postnatal women's leisure time physical activity patterns: a multilevel longitudinal analysis. Res Q Exerc Sport.

[26] Currie S, Sinclair M, Murphy MH, Madden E, Dunwoody L, Liddle D (2013). Reducing the decline in physical activity during pregnancy: a systematic review of behaviour change interventions. PLoS One.

[27] Leiferman J, Swibas T, Koiness K, Marshall JA, Dunn AL (2011). My baby, my move: examination of perceived barriers and motivating factors related to antenatal physical activity. Journal Midwifery Women's Health.

[28] Cioffi J, Schmied V, Dahlen H, Mills A, Thornton C, Duff M (2010). Physical activity in pregnancy: women's perceptions, practices, and influencing factors. Journal midwifery women's health.

[29] Rodgers R.W. Cognitive and physiological processes in fear appeals and attitude change: A revised theory of protection motivation. In: B. L. Cacioppo & L. L. Petty, editor. Social Psychophysiology: A Sourcebook. New York: US: Guilford Press; 1983. p. 153–176.

[30] Smith R, Shakespeare J, Williams Z, Knight M, Foster C (2017). Physical activity for pregnant women: an infographic for healthcare professionals. British Journal of General Practice.

[31] Currie S, Gray C, Shepherd A, McInnes RJ (2016). Antenatal physical activity: a qualitative study exploring women's experiences and the acceptability of antenatal walking groups. BMC Pregnancy Childbirth..

[32] van Mulken MR, McAllister M, Lowe JB (2016). The stigmatisation of pregnancy: societal influences on pregnant women's physical activity behaviour. Culture, health sexuality.

[33] Ferrari RM, Siega-Riz AM, Evenson KR, Moos MK, Carrier KS (2013). A qualitative study of women's perceptions of provider advice about diet and physical activity during pregnancy. Patient Educ Couns.

[34] Duthie E (2013). A, drew E, M, Flynn K, E. patient-provider communication about gestational weight gain among nulliparous women: a qualitative study of the views of obstetricians and first-time pregnant women. BMC Pregnancy Childbirth.

[35] Bennett DL (2017). Bumps and bicycles: Women's experience of cycle-commuting during pregnancy. J Transp Health.

[36] McPhail S, Schippers M (2012). An evolving perspective on physical activity counselling by medical professionals. BMC Fam Pract.

[37] Evenson KR, Wen F (2010). National trends in self-reported physical activity and sedentary behaviors among pregnant women: NHANES 1999–2006. Prev Med.

